# Elastomeric seal stress analysis using photoelastic experimental hybrid method

**DOI:** 10.1038/s41598-022-23568-0

**Published:** 2022-11-21

**Authors:** Bruno R. Mose, Dong-Kil Shin, Bernard O. Alunda, Jeong Hwan Nam

**Affiliations:** 1 School of Mechanical, Manufacturing and Materials Engineering, Jomo Kenyatta University of Agriculture And Technology, Juja, Kenya; 2 School of Mechanical Engineering, Yeungnam University, 280 Daehak-Ro, Gyeongsan-Si, Gyeongsangbuk-Do 38541 South Korea; 3 School of Mines and Engineering, Taita Taveta University, Voi, Kenya; 4 Department of Mechanical System Engineering, Dongyang University, 145 Dongyangdaero, Punggi Yeongju, Yeongju-Si, 750-711 Gyeongbuk Korea

**Keywords:** Engineering, Materials science

## Abstract

Stress freezing is an important and powerful procedure in 3-dimensional experimental stress analysis using photoelasticity. The application of the stress freezing technique to extract stress components from loaded engineering structures has, however, declined over the years even though its principles are well established. This is attributed to huge costs arising from energy consumption during the process. In addition, significant time is needed to generate the desired information from isoclinic and isochromatic fringes. To overcome the limitations of stress freezing in photoelasticity and transform it into an economical device for stress analysis in an engineering environment, a new stress freezing cycle that lasts 5 h is proposed. The proposed technique is used in several applications of elastomeric seals with different cross-sectional profiles to assess their suitability. It was found that reducing the cycle time can lead to huge energy savings without compromising the quality of the fringes. Moreover, the use of isochromatic only to extract stress components leads to a shorter processing time to achieve desirable information since the process of obtaining isoclinic data is involving. In this paper, results of stress analysis from stress-frozen elastomeric seals with various cross-sections using the new stress freezing cycle are presented.

## Introduction

When certain polymeric materials with birefringence capabilities are heated above the glass transition temperature and loaded, then slowly cooled to normal room temperature, the stresses induced by the loads will remain intact even after the load is removed^1,2^. The model specimen made from such polymeric materials with optically sensitive properties can be cut into slices and studied to determine the stress state in the component. This forms the basis of the stress freezing phenomenon, which has been widely used in 3-dimensional photoelasticity for the experimental analysis of stresses in complex structures. For simple geometries, mathematical tools can be used for stress analysis. In real engineering applications, the geometries are complex therefore mathematical models may not be applied. Therefore, 3-dimensional photoelasticity becomes a better tool for predicting stresses in complex engineering structures. This technique has been recognized as an important and powerful method to extract stress components from loaded engineering structures. However, its usage has declined over the years due to huge costs arising from energy consumption during the process. Moreover, significant time is required to generate the desired data from isoclinic and isochromatic fringes.

Drucker^3^ predicted the “imminent death” of photoelastic experimental stress computations and their place taken by computer-based techniques such as the finite element method FEM^4^. The computer-based tools for stress analysis were introduced with a lot of excitement with the capability of allowing researchers to verify results without the need of developing prototypes. Also, they enable simultaneous calculation and visualization of dynamic physical phenomena in addition to the extrapolation of experimental results. However, the accuracy of such tools has come into question especially considering the numerous assumptions made in the formulation of the solutions. Additionally, most computational software is very complex and expensive making it unavailable in the hands of most scientists.

Experimental stress analysts have in recent years rediscovered experimental methods as being more realistic tools and this has led to a re-birth of 3-dimensional photoelasticity^5^. Modern manufacturing technologies such as rapid prototyping (3-D printing) and digital photoelasticity have also made 3-D photoelasticity an attractive alternative tool for stress analysis compared to FEM. The application of 3D-printed photoelastic models containing complex structures has been carried out by various authors^6–10^. 3D printing integrating stress freezing techniques offer great potential which can be used to show the stress field and its evolution in complicated geometries to promote validation of a variety of designs^11^. Although 3-dimensional photoelasticity is steadily being incorporated into the engineering design process, certain challenges need to be overcome.

**Figure 1:**
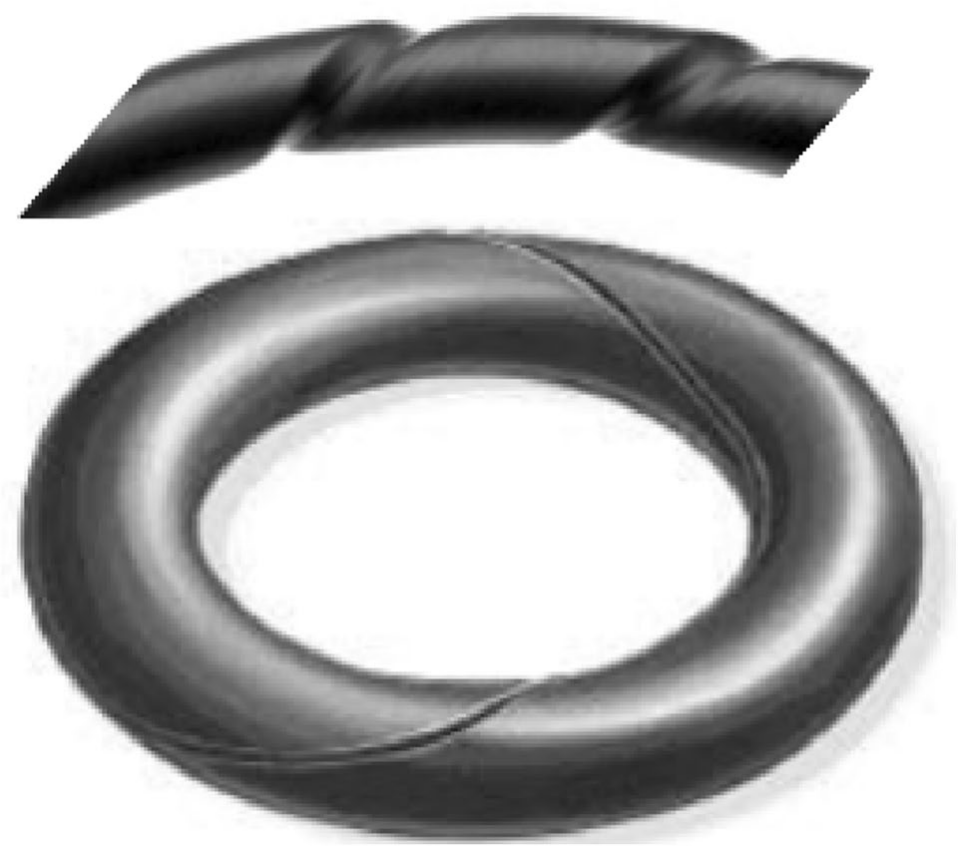
Torsional failure of O-ring.

Over the years, elastomeric seals with O-shaped profiles commonly known as O-rings, have found wide use in sealing applications to control fluid flow under high pressure escaping from one compartment to the other in pumps, boilers, nuclear power plants, heat exchangers, etc.^12^. The O-rings are reported to offer a practical method for the ultimate hydraulic seal. During installation, however, the O-ring may twist. Consequently, the elastomeric O-ring assumes a corkscrew shape which is commonly known as a torsional failure as indicated in Fig. 1.

Other limitations of the O-ring include low performance at high temperatures and high rubbing speeds. Disregarding this limitation may result in poor performance of the seal. However, in circumstances where the design specifications allow, the use of O-rings seals ensures lasting and dependable service.

**Figure 2:**
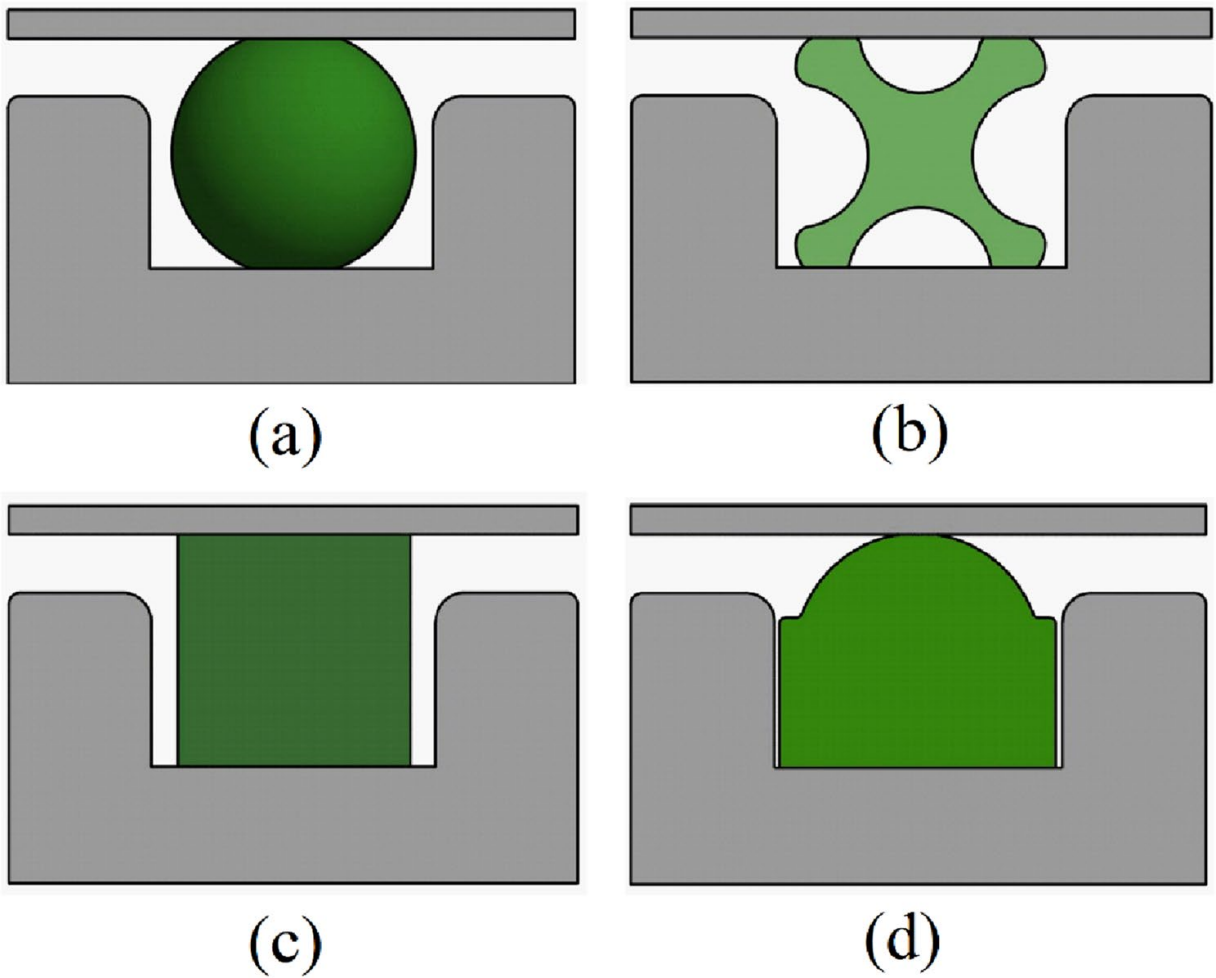
Elastomeric seals with various cross-sectional profiles; (**a**) O-ring (**b**) X-ring (**c**) square ring (**d**) D-ring.

Various cross-sectional profiles have been proposed to minimize torsional failure commonly observed in O-rings Fig. 2a. Some of the profiles that offer alternative and better sealing capabilities are the D-rings, X-rings, and square rings (Fig. 2b–d). The D-ring seal, for instance, has a large and flat geometric base that stops it from twisting and rolling during installation and operation, preventing torsional failure. D-ring seals also offer the advantage that they can be manufactured to standard sizes to replace the O-rings. The X-ring on the other hand has 4 lobes specially designed to improve the lubrication of the seal and prevention of the rolling effect common in O-ring seals. Moreover, the X-rings allow operation in a wide pressure and temperature range and high reciprocating speeds.

In this paper, new stress freezing cycle for photoelastic models of complex geometries is proposed with the advantage of huge savings on energy requirements compared to previously utilized cycles^13–17^. The proposed cycle is applied in stress freezing of elastomeric epoxy resin seals made from Araldite (B41) and cured with Hardener (HT 903) with different cross-sectional profiles to assess its suitability. The cross-sectional profiles considered include the D-ring, square ring, and X-ring. These profiles were selected over the O-ring due to the inherent limitations of O-ring seals.

## Theoretical background

### Hertz contact theory

In a plane problem, the stress components can be easily represented using Muskhelishvilli’s potential functions ξ and ϑ in conjunction with the Airy stress function as shown in Eq. (1)^18^. It is to be noted that the stress components comprise two (2) complex functions ξ(z) and ϑ(z) which if determined then the stress components can be obtained.

1 $$ \begin{aligned} \sigma_{x} &=  Re\left[ {2{\upxi }\left( {\text{z}} \right) - \overline{z}{\upxi }^{^{\prime}} \left( z \right) - \vartheta \left( {\text{z}} \right)} \right] \\ \sigma_{y} &= Re\left[ {2{\upxi }\left( {\text{z}} \right) - \overline{z}{\upxi }^{^{\prime}} \left( z \right) + \vartheta \left( {\text{z}} \right)} \right] \\ \tau_{xy} &= Im\left[ {\overline{z}{\upxi }^{^{\prime}} \left( z \right) + \vartheta \left( {\text{z}} \right)} \right] \\ \end{aligned} $$

From theory, it is assumed that stress functions ξ(z) and ϑ(z) are analytic functions, and can be represented in terms of power series, as shown in Eq. (2).

2a $$ {\upxi }\left( {\text{z}} \right) = \mathop \sum \limits_{n = 0}^{N} P_{n} z^{\frac{n}{2}} $$

2b $$ \vartheta \left( {\text{z}} \right) = \mathop \sum \limits_{n = 0}^{N} Q_{n} z^{\frac{n}{2}} $$

It can be shown that the relative equation between the complex coefficients of the complex function is as shown in Eq. (3).

3 $$ Q_{n} = - \frac{n}{2}P_{n} - \overline{{P_{n} }} $$

Substituting the relative equation between the complex coefficients of the complex function into the stress functions and replacing the resulting stress functions into Eq. (1), Eq. (4) is determined

4 $$ \begin{aligned} \sigma_{x} \left( z \right) = & \mathop \sum \limits_{n = 1}^{N} Re\left\{ {P_{n} \left[ {2R\left( {n,z} \right) - S\left( {n,z} \right)} \right] + \overline{{P_{n} }} R\left( {n,z} \right)} \right\} \\ \sigma_{y} \left( z \right) = & \mathop \sum \limits_{n = 1}^{N} Re\left\{ {P_{n} \left[ {2R\left( {n,z} \right) + S\left( {n,z} \right)} \right] + \overline{{P_{n} }} R\left( {n,z} \right)} \right\} \\ \tau_{xy} = & \mathop \sum \limits_{n = 1}^{N} Im\left\{ {P_{n} S\left( {n,z} \right) - \overline{{P_{n} }} R\left( {n,z} \right)} \right\} \\ \end{aligned} $$

where $$R\left( {n,z} \right) = \frac{n}{2}z^{{\frac{n}{2} - 1}}$$ and $$S\left( {n,z} \right) = \frac{n}{2}\left[ {\left( {\frac{n}{2} - 1} \right)\overline{z} - \frac{n}{2}z} \right] z^{{\frac{n}{2} - 2}}$$.

The beauty of using photoelasticity is that it can quantify stresses throughout a 3D structure and determine the stress gradients however, it requires birefringent material. The maximum and minimum principal stresses are expressed using Eqs. (5a) and (5b) respectively

5a $$ \sigma_{1} = \frac{{\sigma_{x} + \sigma_{y} }}{2} + \sqrt {\left( {\frac{{\sigma_{x} - \sigma_{y} }}{2}} \right)^{2} + \tau_{xy}^{2} } $$

5b $$ \sigma_{2} = \frac{{\sigma_{x} + \sigma_{y} }}{2} - \sqrt {\left( {\frac{{\sigma_{x} - \sigma_{y} }}{2}} \right)^{2} + \tau_{xy}^{2} } $$

### Stress analysis by photoelastic experimental hybrid method

To extract the stress components in a structure under various loading conditions using photoelastic data, it is important to develop mathematical relations between the various stress components, sample specimens, and the optical parameters such as refraction indices, a fringe constant used in photoelasticity. For an isotropic material, Eq. (6) is used to describe the stress optic law^19^.

6 $$ \sigma_{1} - \sigma_{2} = \frac{{N_{f} f_{\sigma } }}{t} $$

By combining Eqs. (5a), (5b), and 6, the stress optic law in Eq. (7) can be easily obtained.

7 $$ \left( {f_{\sigma }^{2} \cdot N_{f}^{2} } \right)t^{ - 2} - \left( {\sigma_{x} - \sigma_{y} } \right)^{2} - 4\tau_{xy}^{2} $$

where *f*_*σ*_ is the fringe value of the stress, *N*_*f*_ is the fringe order, and *t* is the specimen thickness.

In the photoelastic experimental hybrid method, a MATLAB program has been developed by the authors and applied in various studies^13–17^. For the MATLAB program to process the stress components, experimental data such as the specimen thickness, fringe value and fringe order as well as high temperature material properties including Young’s Modulus (E), and Poisson’s ratio (v), are used. When the experimental data, namely the fringe value (*f*_*σ*_), the fringe order (*N*_*f*_), and the specimen thickness (*t*) are substituted into Eq. (7), some errors, *D(ε)*, may arise as shown in Eq. (8) and Eq. (9).

8 $$ \left( {f_{\sigma }^{2} \cdot N_{f}^{2} } \right)t^{ - 2} - \left( {\sigma_{x} - \sigma_{y} } \right)^{2} - 4\tau_{xy}^{2} = D\left( \varepsilon \right) $$

Equation (9) has been obtained when Eq. (4) is substituted into Eq. (8). Equation (9) has been deliberately expanded in power series form to allow the value of *n* to be changed during the iteration process.

9 $$ D\left( \varepsilon \right) = \left( {\frac{{f_{\sigma } N_{f} }}{t}} \right)^{2} - \left\{ {\mathop \sum \limits_{n = 1}^{N} a_{n} Re\left[ {2R\left( {n,z} \right) - 2S\left( {n,z} \right)} \right] + \mathop \sum \limits_{n = 1}^{N} b_{n} Im\left[ {2R\left( {n,z} \right) + 2S\left( {n,z} \right)} \right]} \right\}^{2} - \left\{ {\mathop \sum \limits_{n = 1}^{N} a_{n} Im\left[ {2S\left( {n,z} \right) - 2R\left( {n,z} \right)} \right] + \mathop \sum \limits_{n = 1}^{N} b_{n} Re\left[ {2R\left( {n,z} \right) + 2S\left( {n,z} \right)} \right]} \right\}^{2} $$

In the photoelastic experimental hybrid method, minimizing the errors, D*(ε)* in Eq. (9) is crucial in order to increase the accuracy of the method. The starting point is the selection of the region from which to extract approximately 1000 data points on the *x.0* or *x.5* fringe orders on the isochromatics using imaging software and transferring the data to an excel file. The file captures, in addition, the material properties, specimen geometry and is then sent to MATLAB program for processing. The program picks all the data in Eq. (9) and processes the graphical isochromatic fringes. In case the graphical isochromatics are not similar to the actual isochromatics, it means errors D*(ε)* are large and *n* has to be changed iteratively.

Various numerical tools have been recommended to minimize errors. In the present study, the Hook-Jeeves numerical method was used^20^. When the errors reach the limiting condition of D*(ε)* ≤ 10^–5^, the similarity between the actual and regenerated isochromatics was observed and the program terminated.

The stress functions ξ(z) and ϑ(z) can then be evaluated by plugging in the values of *a*_*n*_ and *b*_*n*_ in the respective equation. When the determined ξ(z) and ϑ(z) are substituted into Eq. (1), then it is possible to obtain the stress components ${\sigma }_{X}$*,* ${\sigma }_{Y}$ and ${\tau }_{XY}$ that is developed in a given structural member when an arbitrary load is applied to it. The prescribed procedures are what comprise the photoelastic experimental hybrid method.

Although other methods such as the phase shifting method, RGB method, and the continuously loading method have been widely used to analyse fringes, they suffer various limitations. For instance, the mentioned methods require thinning algorithms. These algorithms are complex in nature and time-consuming. Moreover, thinning algorithms tend to fail in zones of high stress concentrations^21^.. As a result, new algorithms are being explored to fix these challenges. In recent years, photoelastic experimental hybrid method (PEHM), which is a fringe analysis method has been widely used without the need for thinning algorithms.

## Experiment and experimental method

### Fabrication of photoelastic models

**Figure 3:**
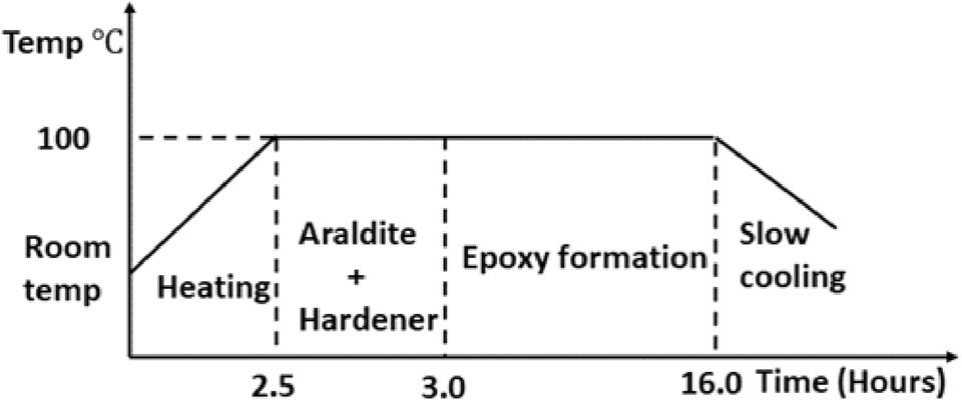
Epoxy resin casting cycle.

The casting of the photoelastic models of the elastomeric seals with the cross-sectional profiles under investigation was done using Araldite (B41, Ciba-Geigy) and hardener (HT 903, Ciba-Geigy) at the ratio of 10:3 following the procedure developed earlier by Nam et al.^13^. The epoxy resin casting cycle is shown in Fig. 3. The epoxy resin was introduced by Leven^22^ as a photoelastic material and they offer great impetus to photoelastic stress analysis compared to other resins such as phenolics resins, styrene alkyd, etc. due to numerous advantages. The epoxy resins come closest to satisfying the requirements of ideal photoelastic materials owing to their ease of machinability, linear stress/fringe relation, shorter curing time, and lack of residual stresses. The epoxy resin is also easy to cast into large sizes, transparent, highly resistant to creep and has both mechanical and optical isotropy. The Araldite resin has been confirmed to be a convenient material for making birefringent models compared to its counterparts such as Flexible GIV, and PL2^23^.

**Figure 4:**
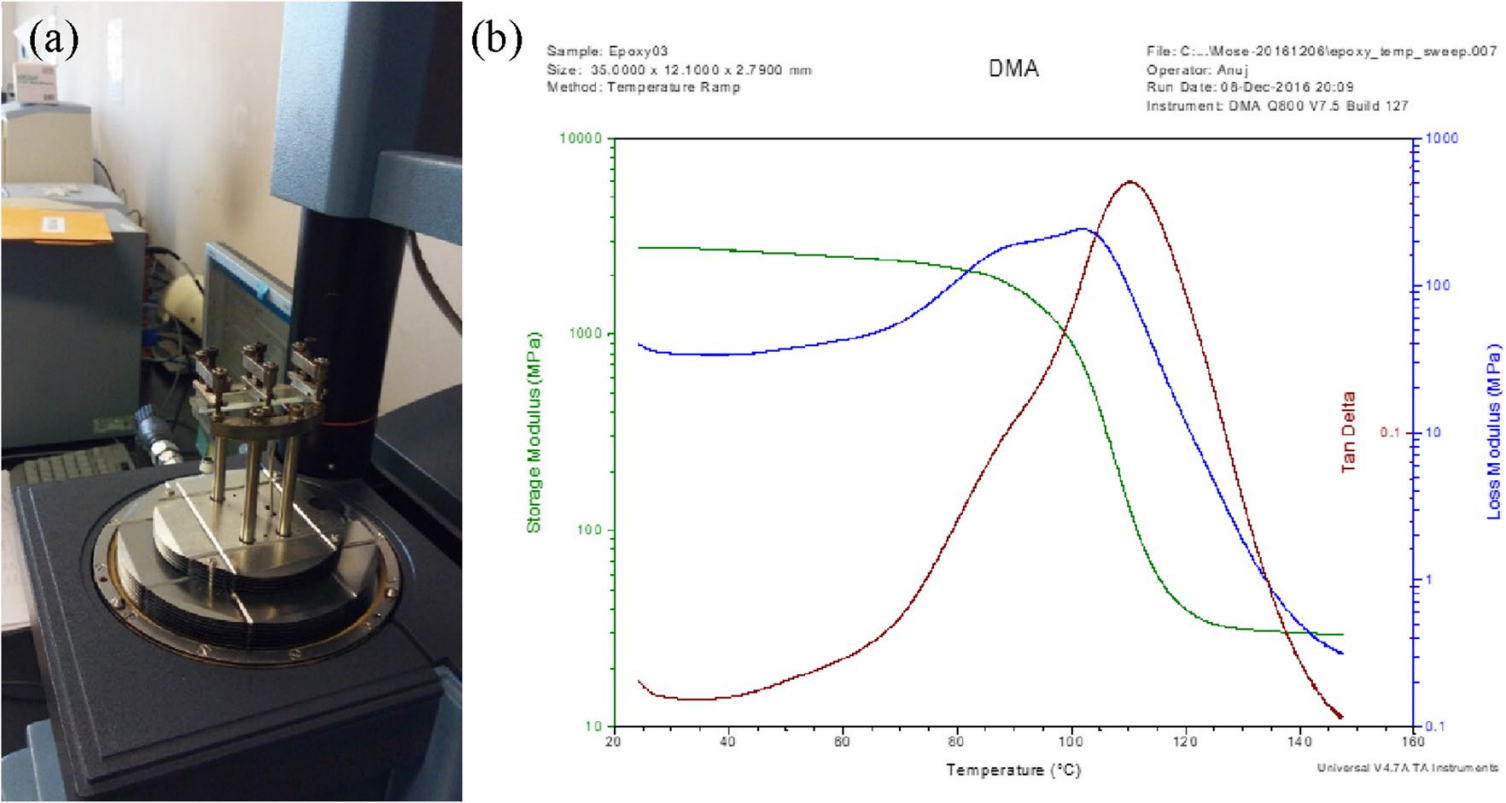
(**a**) DMA experimental set-up for three-point bending test at 1.0 Hz frequency in the temperature range from 25 °C to 150 °C and (**b**) thermo-optic curve of the photoelastic material showing the glass transition temperature^24^.

The presence of residual stress in the fabricated photoelastic models is normally tested visually using a polariscope available in the lab as one of the procedures before loads are applied. The material properties of this epoxy resin have been measured at a stress freezing temperature of 120℃. These properties are; Elastic modulus, E = 15.6 MPa, Poisson’s ratio, ν = 0.47 and stress fringe value, f_σ_ = 242.58 N/m. Thermo-mechanical properties for the fabricated photoelastic models had been determined by the researchers as shown in Fig. 4^24^. It is shown that the photoelastic material used in this research had a glass transition temperature, T_g_ of 110 °C.

### Stress freezing cycle

In a two-dimensional photoelastic experimental analysis^25,26^, a suitable model made from either epoxy or other materials is fabricated, and loaded while it is inside the photoelastic experimental rig and the fringe pattern examined is photographed which is later interpreted. However, in a real situation especially in industries, complicated problems exist which are 3-dimensional and so the stress analysis of such problems requires special techniques. One of the techniques employed is the stress freezing method which permits the construction of the desired model, loading, it appropriately and later analyzing the interior planes of the model using photoelasticity^27^. In the stress freezing method, the deformations of the model and the related optical responses are locked into the loaded 3-dimensional model. The model is then sliced, polished, and positioned on the loading device of a transparent photoelastic experimental device to acquire the interior stress information.

The pioneering work of the stress freezing method was an initiative of Oppel^28^, Kuske^29^, Hetenyi^30^, and Frocht^31^ in Germany way back in 1936 but its usage was limited especially in the industry because its was time-consuming and so expensive though it was such a powerful tool. In his research, Jan Cernosek^2^ showed the competitive and cost-effectiveness of three-dimensional photoelasticity.

**Figure 5:**
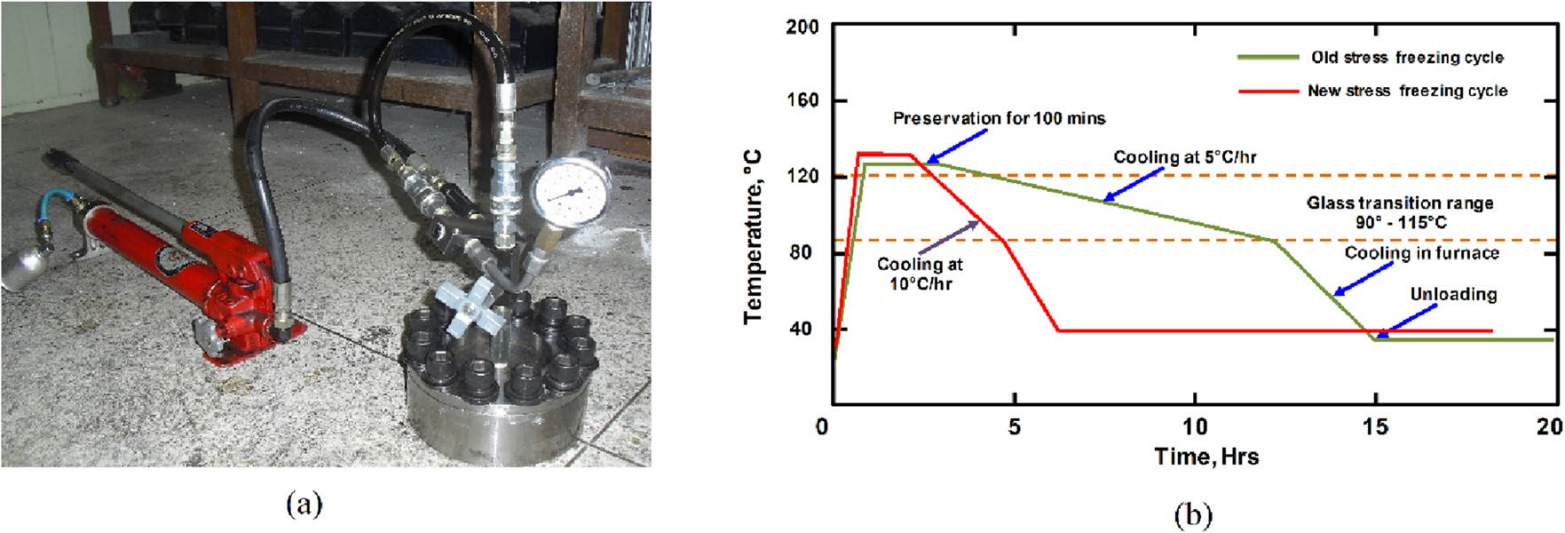
(**a**) Loading equipment capable of subjecting the seals to uniform compression and internal pressure and (**b**) stress freezing cycles (old -Alunda et. al^32^ and new proposed cycle.

The experimental apparatus mainly consists of experimental equipment, specially designed to provide the seals with different geometries with a constant compression rate and varying internal pressure. The experimental equipment was designed with an opening at the top where the internal pressure can be applied using a hydraulic pump as shown in Fig. 5a. In addition, the device has a valve to allow the opening and closing during the application or release of the internal pressure to the seal.

The old stress freezing cycle consisted of consistently heating the specimen to a temperature above the glass transition temperature of the epoxy (usually above 120 °C) before loading (application of the internal pressure), holding it there for approximately 120 min before cooling it at a rate of 5 °C /hr up to a temperature of about 90˚C before switching off and allowing the whole assembly to cool inside the furnace to room temperature. The whole process of stress freezing would require the furnace to run for at least 12 h which was time-consuming and expends a lot of energy.

In the new stress freezing method, various seals with different cross-sectional profiles were installed in the photoelastic experimental rig and compressed with a uniform squeeze rate of 20% followed by the application of the various internal pressures. The experimentation was carried out using the newly proposed stress freezing cycle that involved the following steps;

1. The seals fabricated from epoxy resin and the experimental equipment were placed in the furnace and separately heated for roughly one (1) hour at a constant temperature of 125 °C (just above the end of the glass transition temperature,) in the stress-freezing furnace so that the epoxy resin attains the glass transition temperature, *T*_*g*_.
2. The seals (one at a time), and the photoelastic experimental rig were assembled inside the furnace to impact the required compression rate and internal pressure.
3. The assembled photoelastic experimental rig was heated at that temperature for 120 min before being allowed to cool at a rate of 10˚C/hr up to the beginning of the glass transition temperature.
4. The assembled photoelastic experimental rig was permitted to cool to ambient temperature inside the furnace.

The old and new stress freezing cycles were plotted in the same graph as shown in Fig. 5b for comparison. The new freezing cycle required the furnace to operate for about 5 h only.

**Figure 6:**
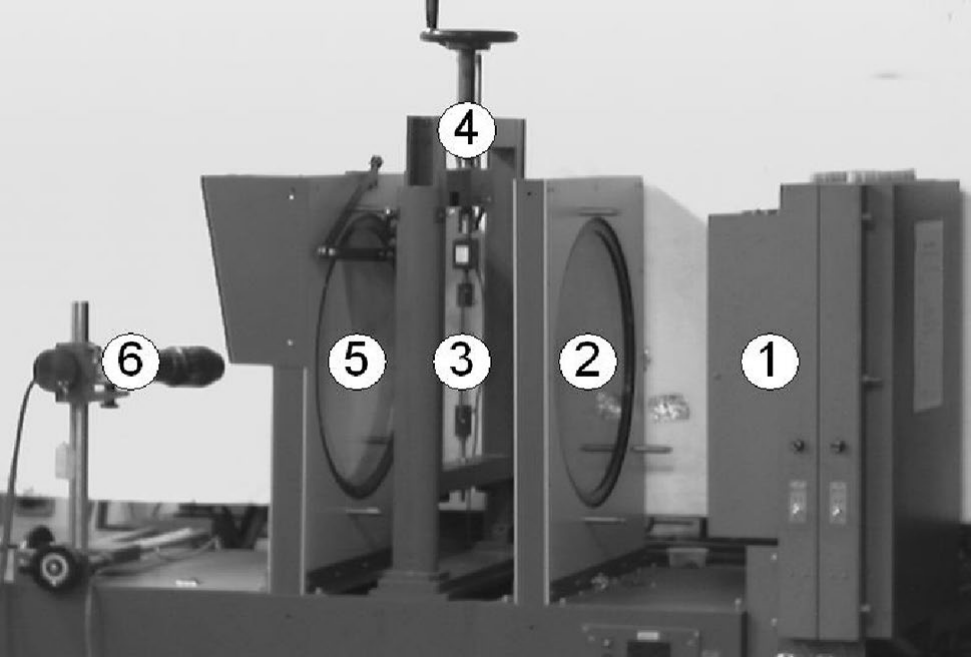
Schematic of transparent photoelastic experiment device. ① - Light source, ② - polarizer/1st quarter-wave plate, ③ - specimen, ④ - loading device, ⑤ - analyzer/2nd quarter-wave plate ⑥ - high-speed camera.

After stress freezing, the seals with different geometries were sliced into thin sections for further analysis using the photoelastic experimental device shown in Fig. 6. The photoelastic experimental device is composed of a light source for illumination (No. 1) followed by the polarizer/1st quarter-wave plate (No. 2). The specimen (No. 3) is positioned amid the polarizer/1st quarter-wave plate (No. 2) and the analyzer/2nd quarter-wave plate (No. 5). To complete the photoelasticity system the high-speed camera (No. 6) is provided to capture the images. Usually, the two (2) quarter-wave plates are added to polarizer and analyzer to achieve circularly polarized light that is capable of only producing the isochromatic fringes alone and not the isoclinic fringes. This allows for easy discrimination of the isoclinic and the isochromatic fringe patterns. The thin slices for analysis were cut using a saw and polished properly to a minimum thickness of about 0.8 mm. To achieve good-quality digital images, the polished slices were put in a box of glass filled with a mixed solution of organic alcohol and paraffin before positioning on the transparent photoelastic experimental rig. A high-speed digital CCD camera (No. 6) was then used to capture the isochromatic fringe patterns and then moved to a personal computer for further analysis.

## Experimental results and discussion

**Figure 7:**
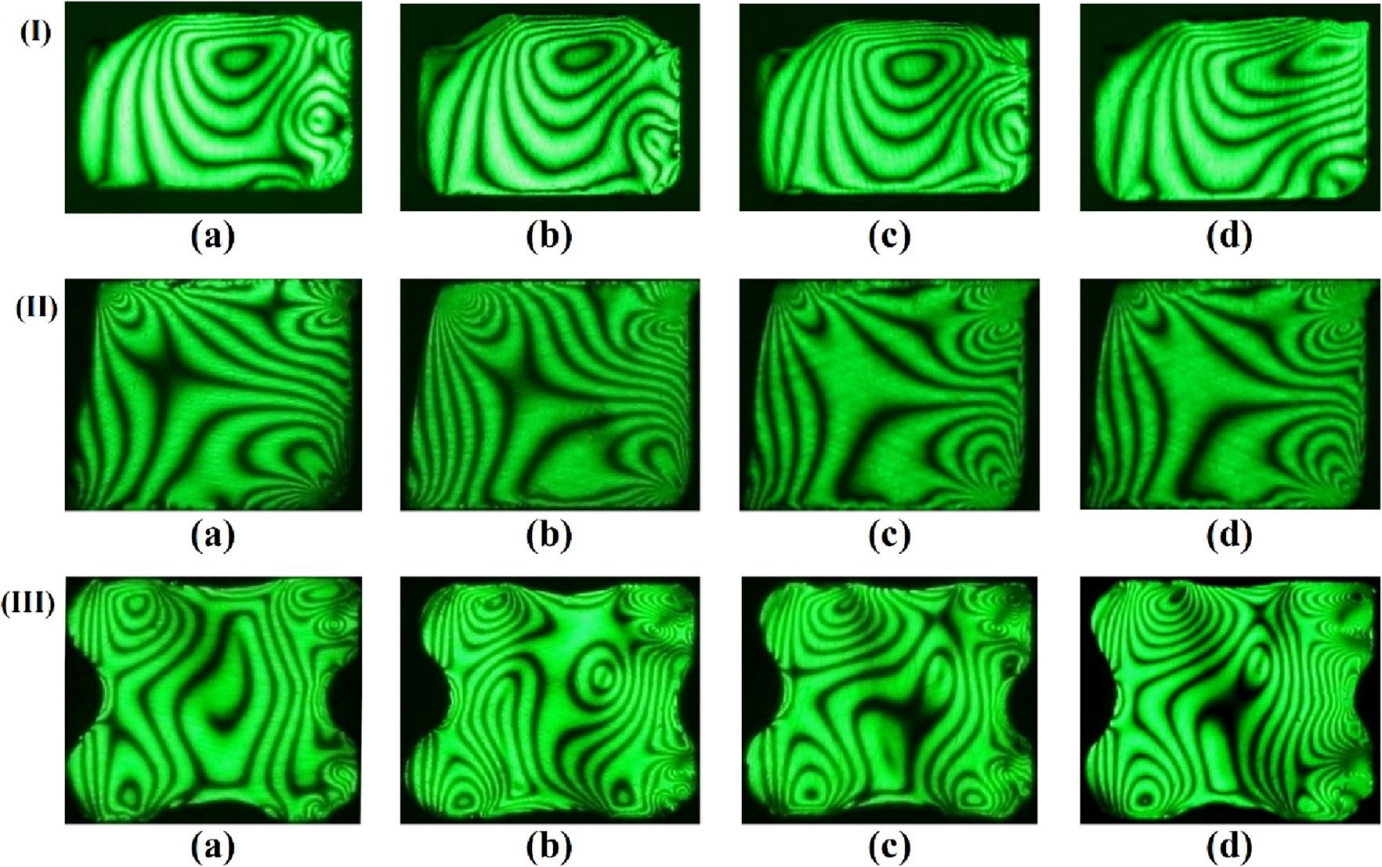
The isochromatic fringe patterns of (I) D-ring, (II) square ring, and (III) X-ring were obtained using the photoelastic experimental rig at different internal pressures. (**a**) Pi =1.0 MPa (**b**) Pi =2.0 MPa (**c**) Pi =3.0 MPa (**d**) Pi =4.0 MPa.

The effectiveness of the proposed stress freezing cycle to yield good quality isochromatic fringes for further analysis was evaluated. It can be seen from Fig. 7 that the cycle resulted in good quality isochromatic fringes which are very clear. The isochromatic fringe patterns are distinguishable and increase in number as one approaches the contact zones.

**Figure 8:**
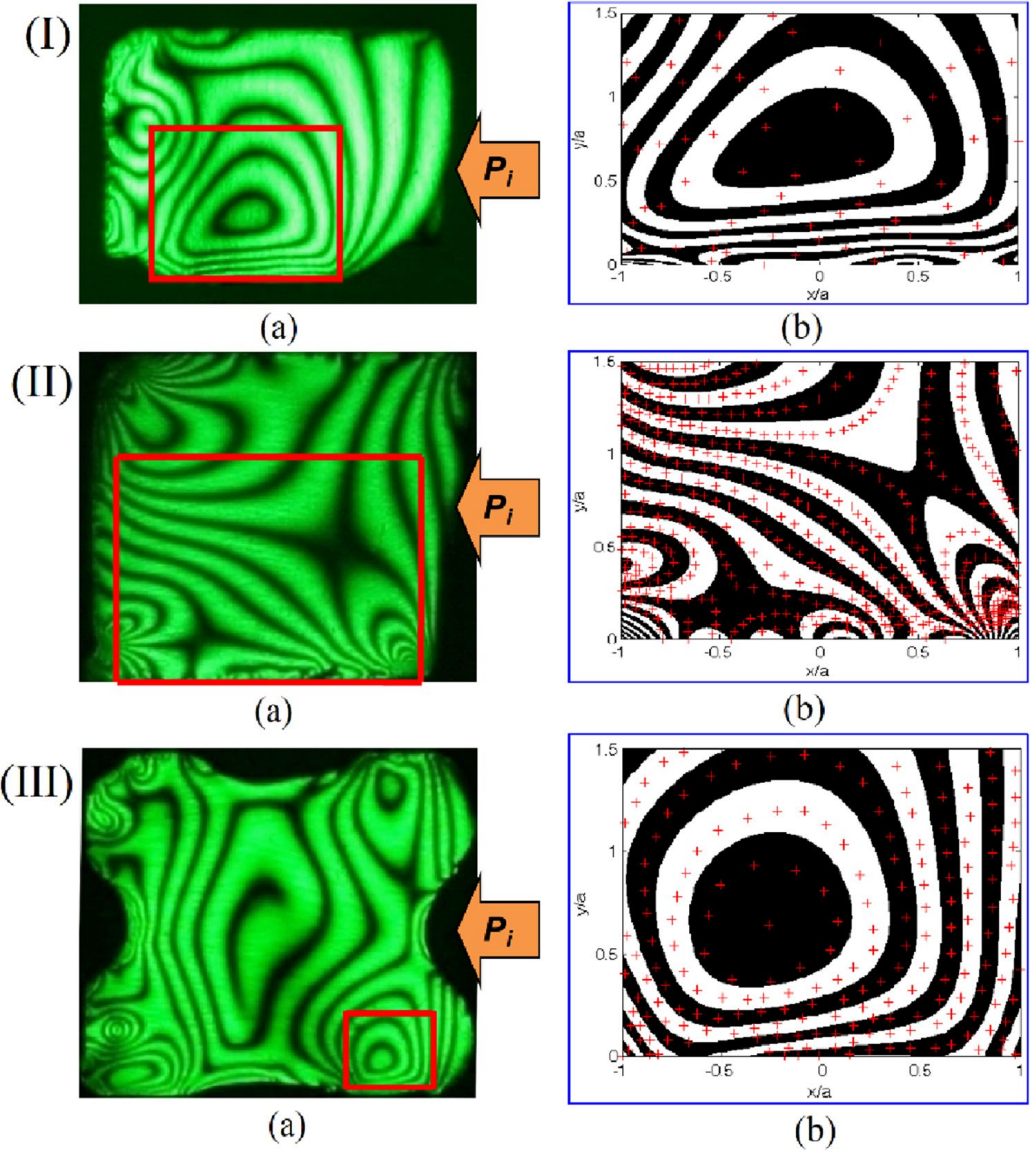
The actual (**a**), and the graphical (**b**) isochromatic fringe patterns on the upper side of; (**I**) D-ring, (**II**) Square ring, and (**III**) X-ring from the photoelastic experimental hybrid method under a uniform compression of 20% and internal pressure (*P*_*i*_=1 MPa).

A representative analysis of the contact stresses was done for each seal geometry to ascertain the distribution of stresses on the upper surface under a uniform compression of 20% and internal pressure of 1.0 MPa. The photoelastic experimental hybrid method was employed in the analysis of the stresses of the contact problem. The required data from the experiment for the photoelastic experimental hybrid was obtained from the highlighted quadrilateral box (□) in the actual isochromatic fringe pattern (Fig. 8). The “ + ” marks used in the actual isochromatic show the positions from which the experimental data was gathered. The cross marks “ + ” used are assumed to be located at the center of each of the white or dark bands with little permitted errors. Collected experimental data together with material properties serve as input data into a MATLAB program developed by the authors. Through an iteration program the component stresses are obtained when there is indistinguishability between the actual and graphic isochromatic as shown in Fig. 7. Thus, through this procedure, it can be deduced that the contact and interior stresses match well with those of the actual specimen under consideration.

**Figure 9:**
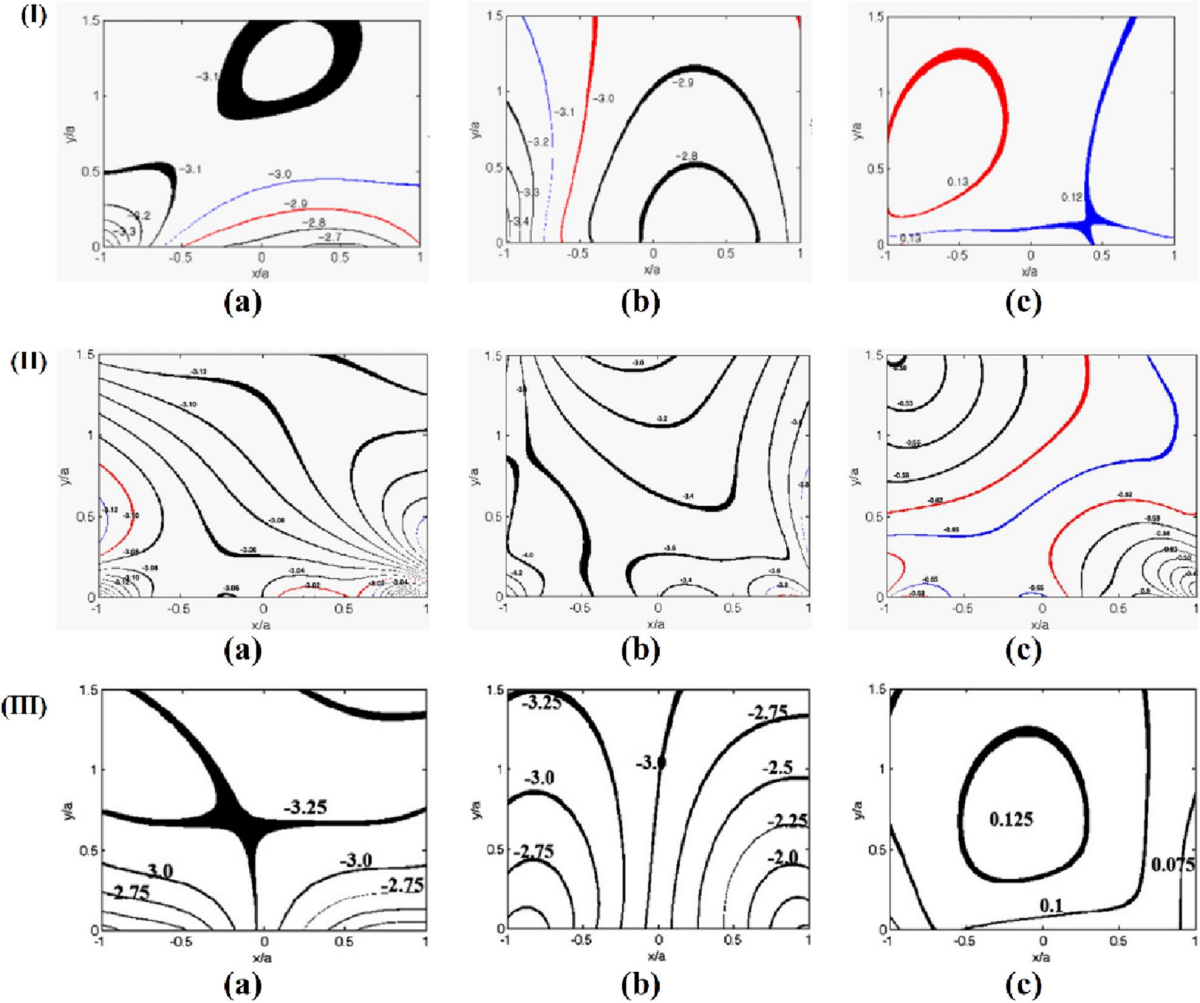
The internal stress contours on the upper region of; (I) D-ring, (II) Square ring, and (III) X-ring under uniform compression of 20% normalized by internal pressure (*P*_*i*_) of 1 MPa. (a), (b), and (c) represents *σ*_*X*_*/P*_*i*_, *σ*_*Y*_*/P*_*i*_, and *τ*_*XY*_*/P*_*i*_ respectively.

**Figure 10:**
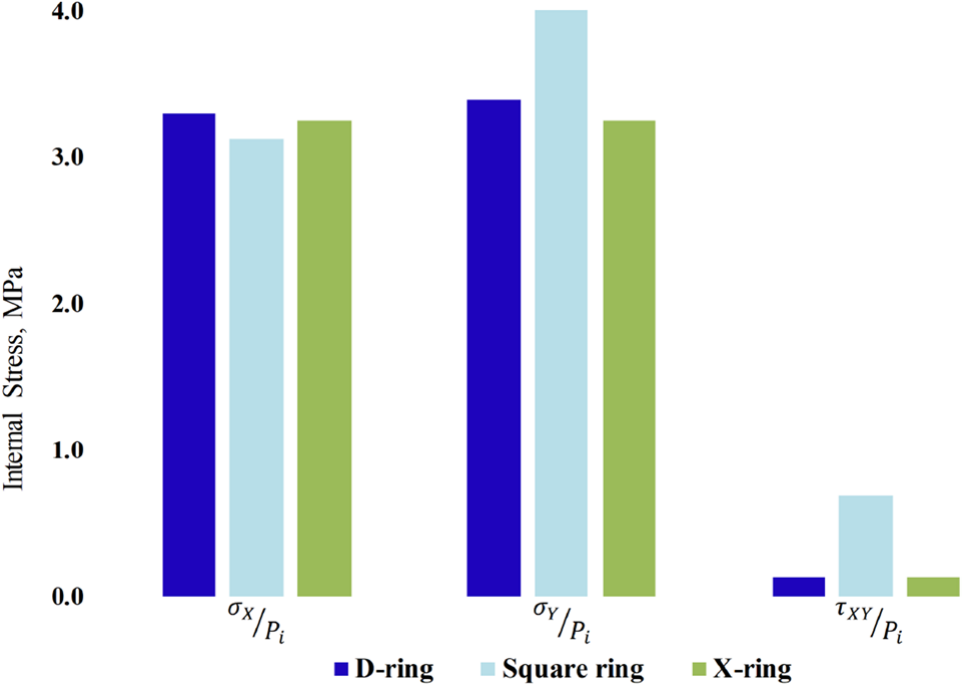
A comparison of the performance of various seals with pressure applied.

The internal stress contours of the respective seal geometries were also generated as shown in Fig. 9. *P*_*i*_ denotes the internal pressure used to normalize the internal stresses and the contact stresses. For the D-ring and square-rings, the normalized *σ*_*X*_, *σ*_*Y*_, and *τ*_*XY*_ internal stresses were 3.3 MPa, 3.4 MPa, 0.13 MPa, and 3.12 MPa, 4.2 MPa, and 0.68 MPa respectively. Also, the highest magnitudes of the normalized internal stresses *σ*_*X*_, *σ*_*Y*_, and *τ*_*XY*_ for the X-ring were examined and found to be 3.25 MPa, 3.25 MPa, and 0.125 MPa respectively. A summary of the highest internal stresses for the three seal geometries is summarized in the bar graph Fig. 10. The high magnitudes of shear stress computed from the square ring indicate that it is more likely to fail compared to the X-ring and D-ring.

## Conclusion

In this study, a novel stress freezing cycle that lasts 5 h instead of 15 h was proposed and its effectiveness was investigated using the photoelastic experimental hybrid method. It was found that;

1. The proposed stress freezing cycle can yield good-quality isochromatic fringes for experimental stress analysis.
2. The huge costs arising from energy consumption can be saved by the use of the proposed stress freezing cycle
3. The square ring experienced the highest shear stress compared to the D-ring and X-ring. This means that the square ring is more likely to fail since shear stress is considered in engineering stress models as the most damaging as it controls crack initiation
4. The process of extraction of stress components *σ*_*X*_, *σ*_*Y*_, and *τ*_*XY*_ from isochromatic fringes provides a more realistic tool for stress analysis

## Data Availability

The datasets used and/or analyzed during the current study available from the corresponding author on reasonable request.
